# Supplementary material for: Antifungal Activity of Mexican Propolis on Clinical Isolates of Candida Species
Source: Molecules. 2022 Sep 1;27(17):5651. doi: 10.3390/molecules27175651 (PMC9457601; doi:10.3390/molecules27175651)
Supplement: Supplementary file 1 [file molecules-27-05651-s001.zip › molecules-1874466-supplementary.pdf]

SUPPLEMENTARY MATERIAL

INDEX- Supplemental Material

1. **Spectral data**……………………………………………………………………………..2

Figure S1. ^{1}H NMR spectrum of the methanol extract of propolis (700 MHz, DMSO-d6)………….2

Figure S2. ^{1}H NMR spectrum of F40 obtained from fractionation of the methanol extract of propolis (700 MHz, DMSO-d6)……………………………………………………………………...…3

Figure S3. ^{13}C NMR spectrum of F40 obtained from fractionation of the methanol extract of propolis (150 MHz, DMSO-d6)…………………………………………………………………………...4

Figure S4. ^{1}H NMR spectrum of F75 obtained from fractionation of the methanol extract of propolis (700 MHz, DMSO-d6)…………………………………………………………………………...5

Figure S5. ^{13}C NMR spectrum of F75 obtained from fractionation of the methanol extract of propolis (150 MHz, DMSO-d6)…………………………………………………………………………...6

Figure S6. ^{1}H NMR spectrum of F80 obtained from fractionation of the methanol extract of propolis (700 MHz, acetone-d6)…………………………………………………………………………...7

Figure S7. ^{13}C NMR spectrum of F80 obtained from fractionation of the methanol extract of propolis (150 MHz, acetone-d6)…………………………………………………………………………...8

Figure S8. ^{1}H NMR spectrum of F101 obtained from fractionation of the methanol extract of propolis (700 MHz, DMSO-d6)…………………………………………………………………………...9

Figure S9. ^{13}C NMR spectrum of F101 obtained from fractionation of the methanol extract of propolis (150 MHz, DMSO-d6)……………………………………………………………………………10

Figure S10. ^{1}H NMR spectrum of F130 obtained from fractionation of the methanol extract of propolis (600 MHz, Acetone-d6)……………………………………………….………………………..11

Figure S11. ^{13}C NMR spectrum of F130 obtained from fractionation of the methanol extract of propolis (150 MHz, Acetone-d6)…………………………………………………………………..12

Figure S12. ^{1}H NMR spectrum of F160 obtained from fractionation of the methanol extract of propolis (600 MHz, Acetone-d6)……………………………………………….……………………..…13

Figure S13. ^{13}C NMR spectrum of F160 obtained from fractionation of the methanol extract of propolis (150 MHz, Acetone-d6)…………………………………………………………………..14

Figure S14. HRMS (ESI-negative mode) spectra of the methanol extract of propolis…….….……15

Figure S15. HRMS (ESI-negative mode) spectra of F40…………………………………….………16

Figure S16. HRMS (ESI-negative mode) spectra of F80…………………………………….………17

Figure S17. HRMS (ESI-negative mode) spectra of F101……………………………….…………..18

Figure S18. HRMS (ESI-negative mode) spectra of F130…………………………….……………..19

Figure S19. HRMS (ESI-negative mode) spectra of F160…………………………….……………..20

1. Spectral Data

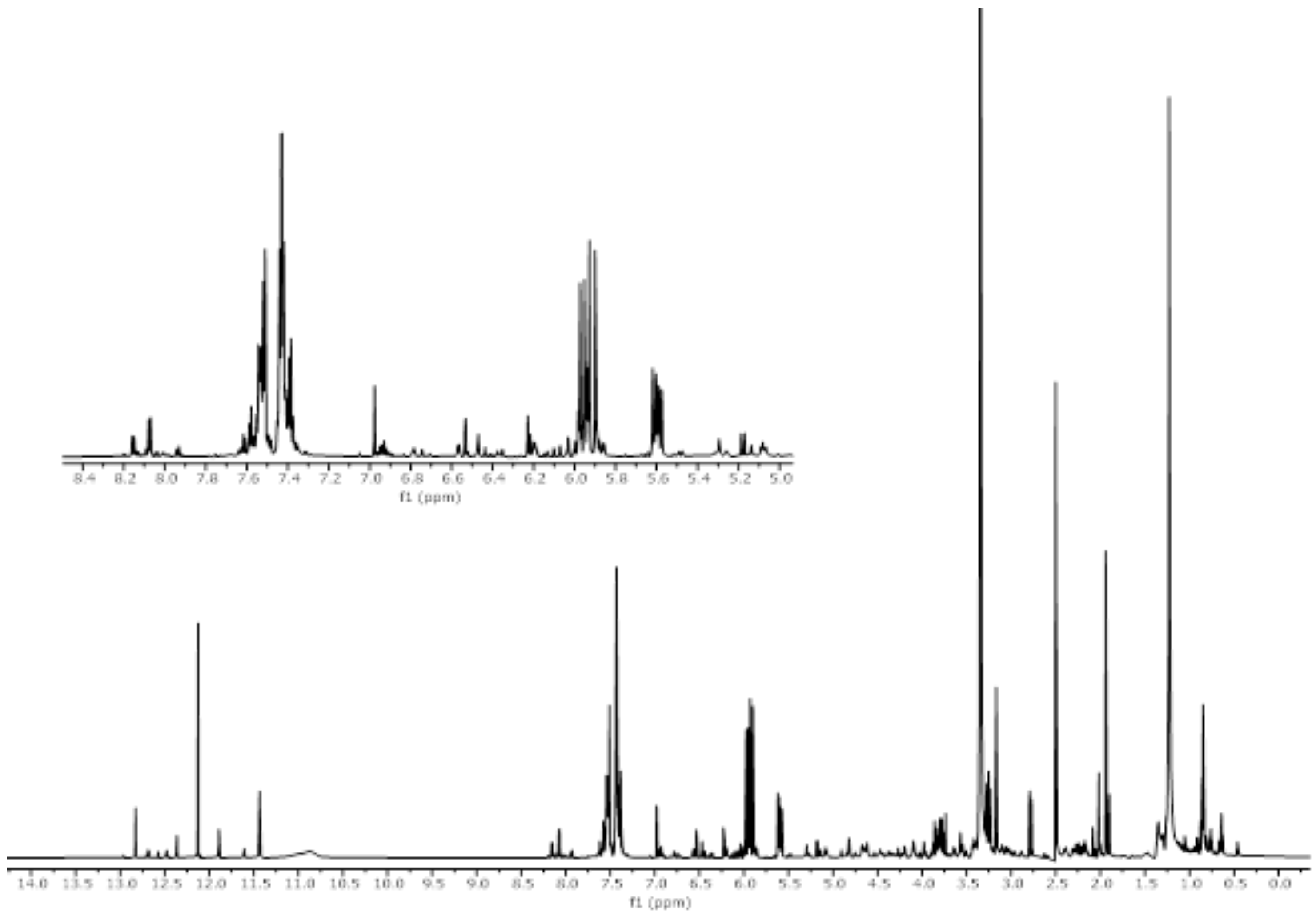

Figure S1. ^{1}H NMR spectrum of the methanol extract of propolis (700 MHz, DMSO-d6).

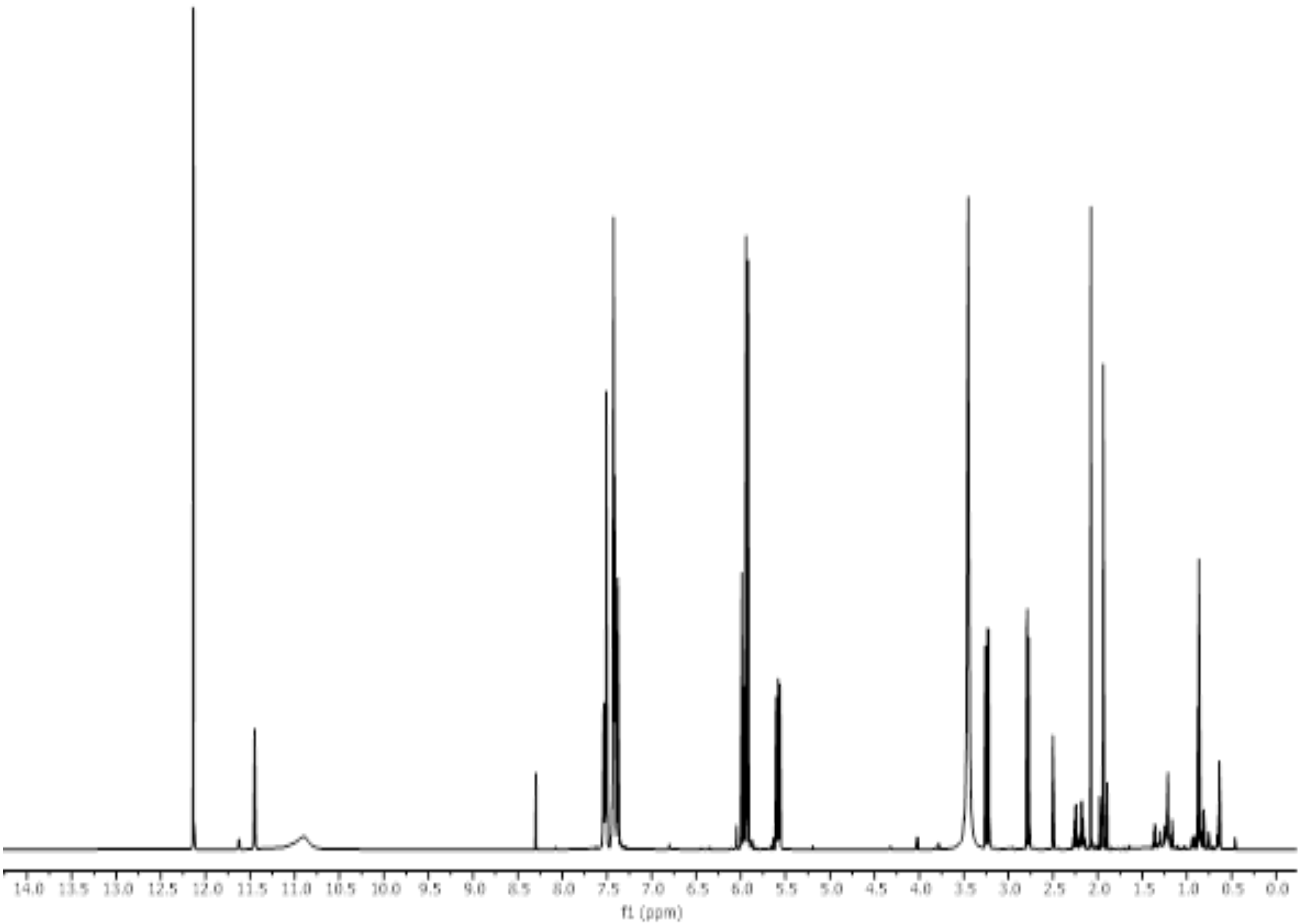

Figure S2. ^{1}H NMR spectrum of F40 obtained from fractionation of the methanol extract of propolis (700 MHz, DMSO-d6).

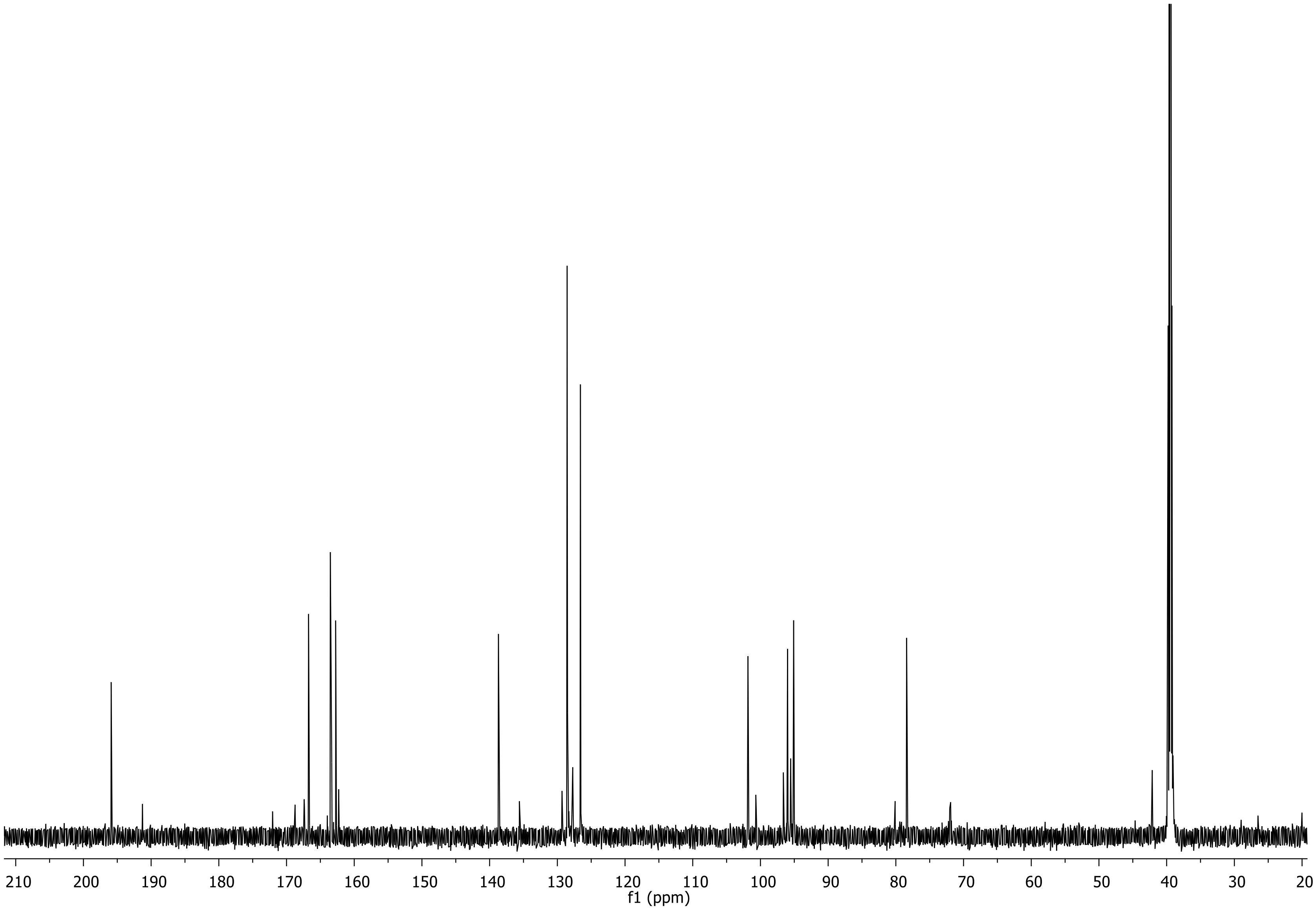

Figure S3. ^{13}C NMR spectrum of F40 obtained from fractionation of the methanol extract of propolis (150 MHz, DMSO-d6).

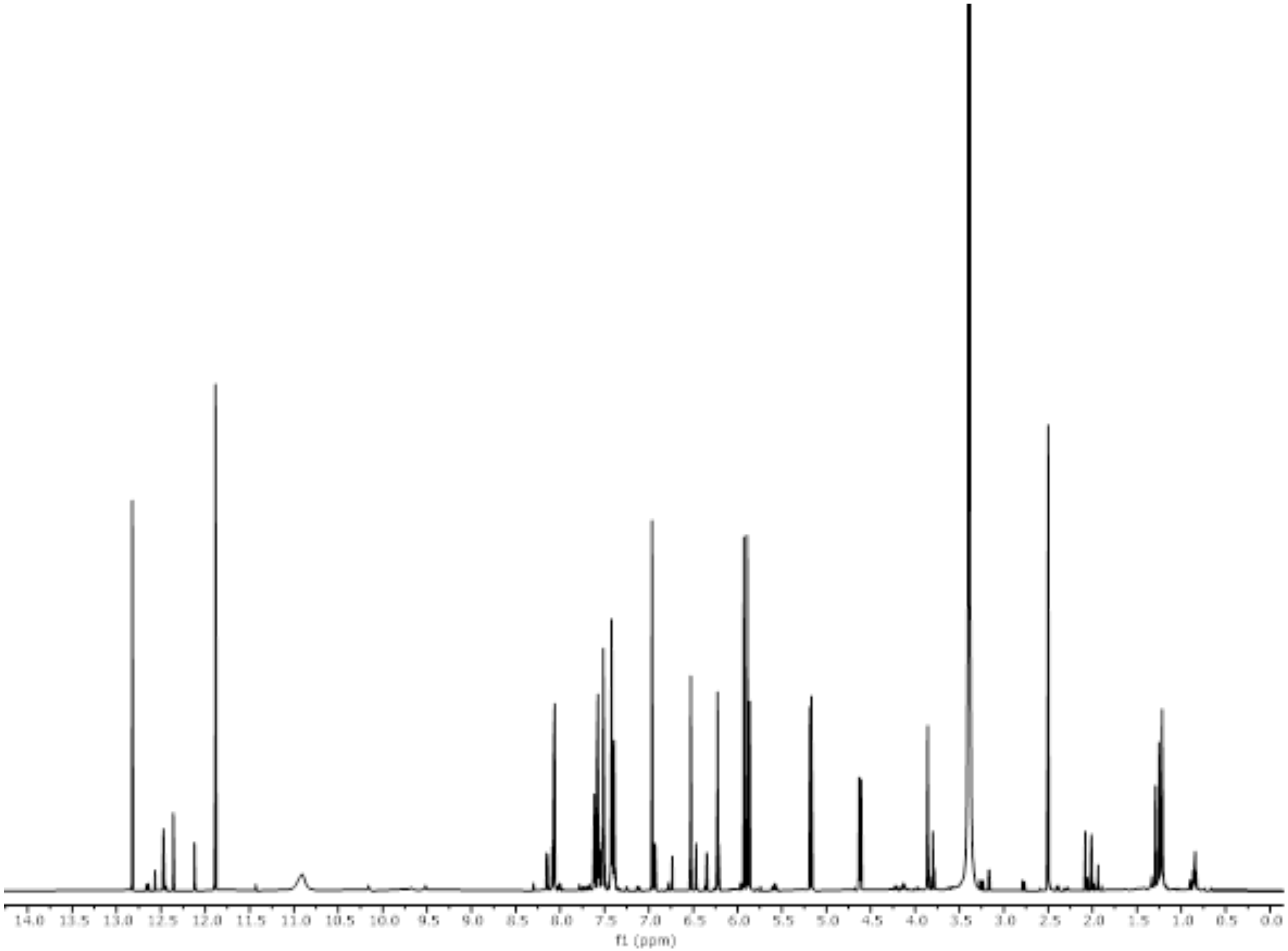

Figure S4. ^{1}H NMR spectrum of F75 obtained from fractionation of the methanol extract of propolis (700 MHz, DMSO-d6).

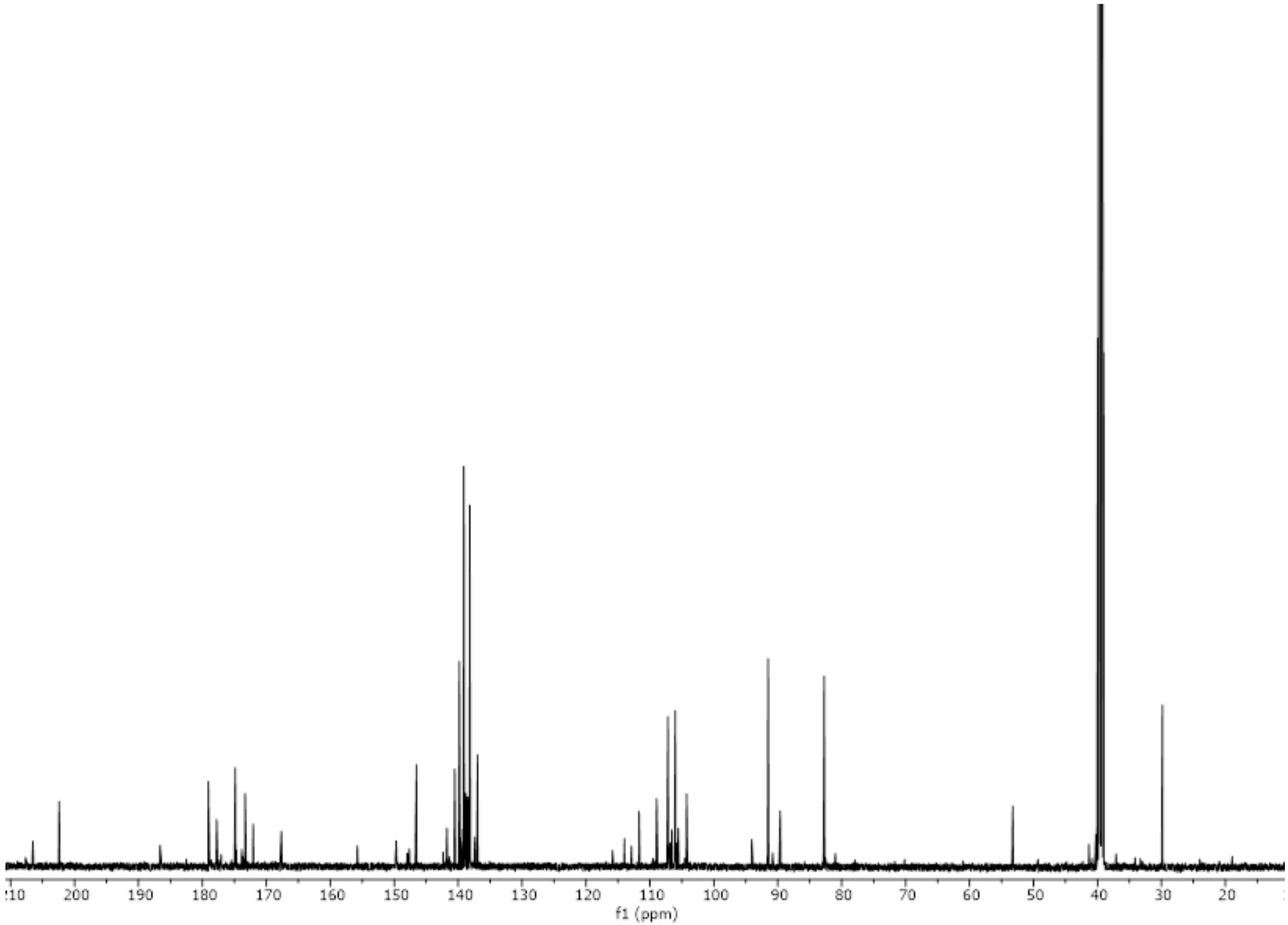

Figure S5. ^{13}C NMR spectrum of F75 obtained from fractionation of the methanol extract of propolis (150 MHz, DMSO-d6).

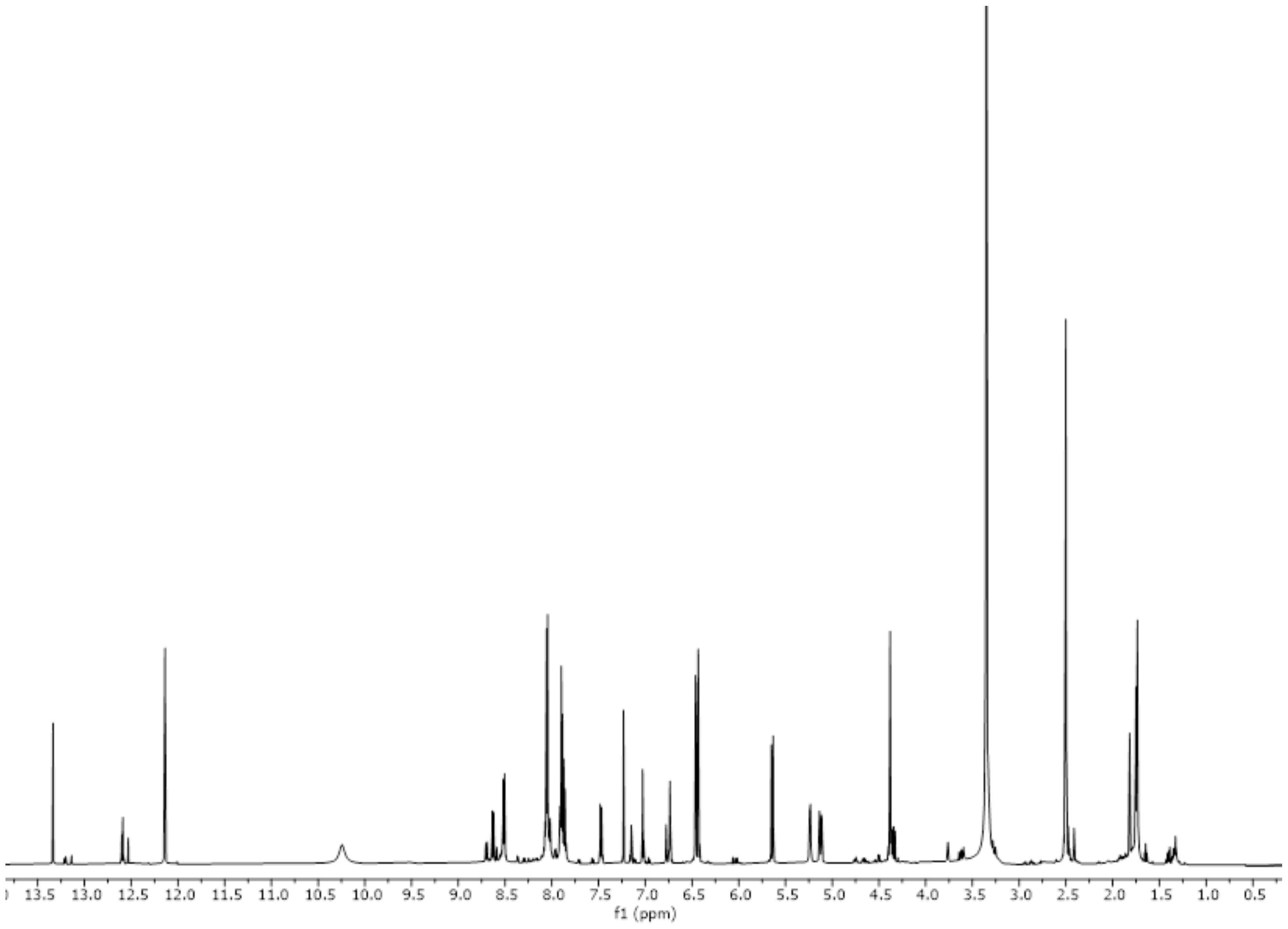

Figure S6. ^{1}H NMR spectrum of F80 obtained from fractionation of the methanol extract of propolis (700 MHz, acetone-d6).

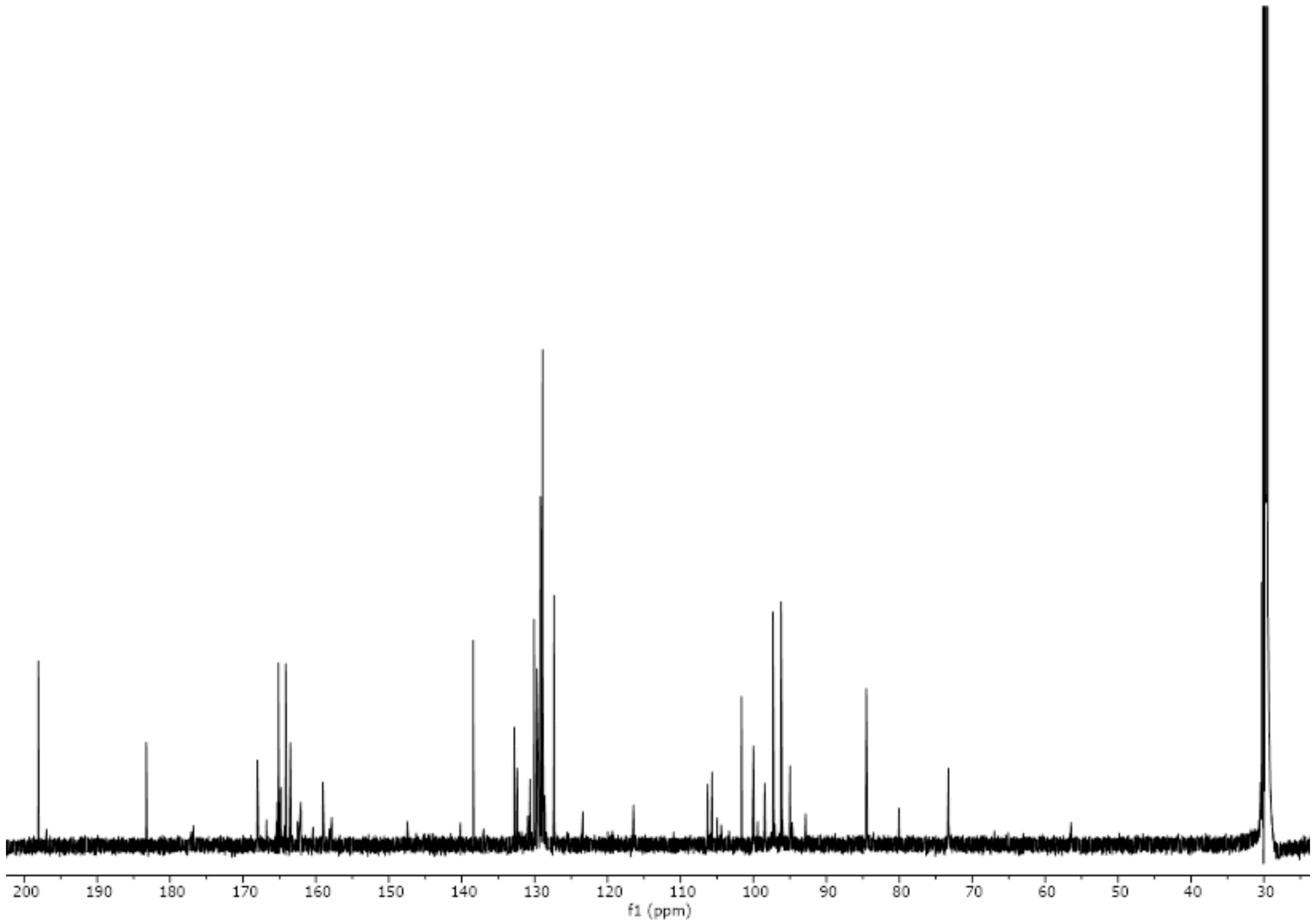

Figure S7. ^{13}C NMR spectrum of F80 obtained from fractionation of the methanol extract of propolis (150 MHz, acetone-d6).

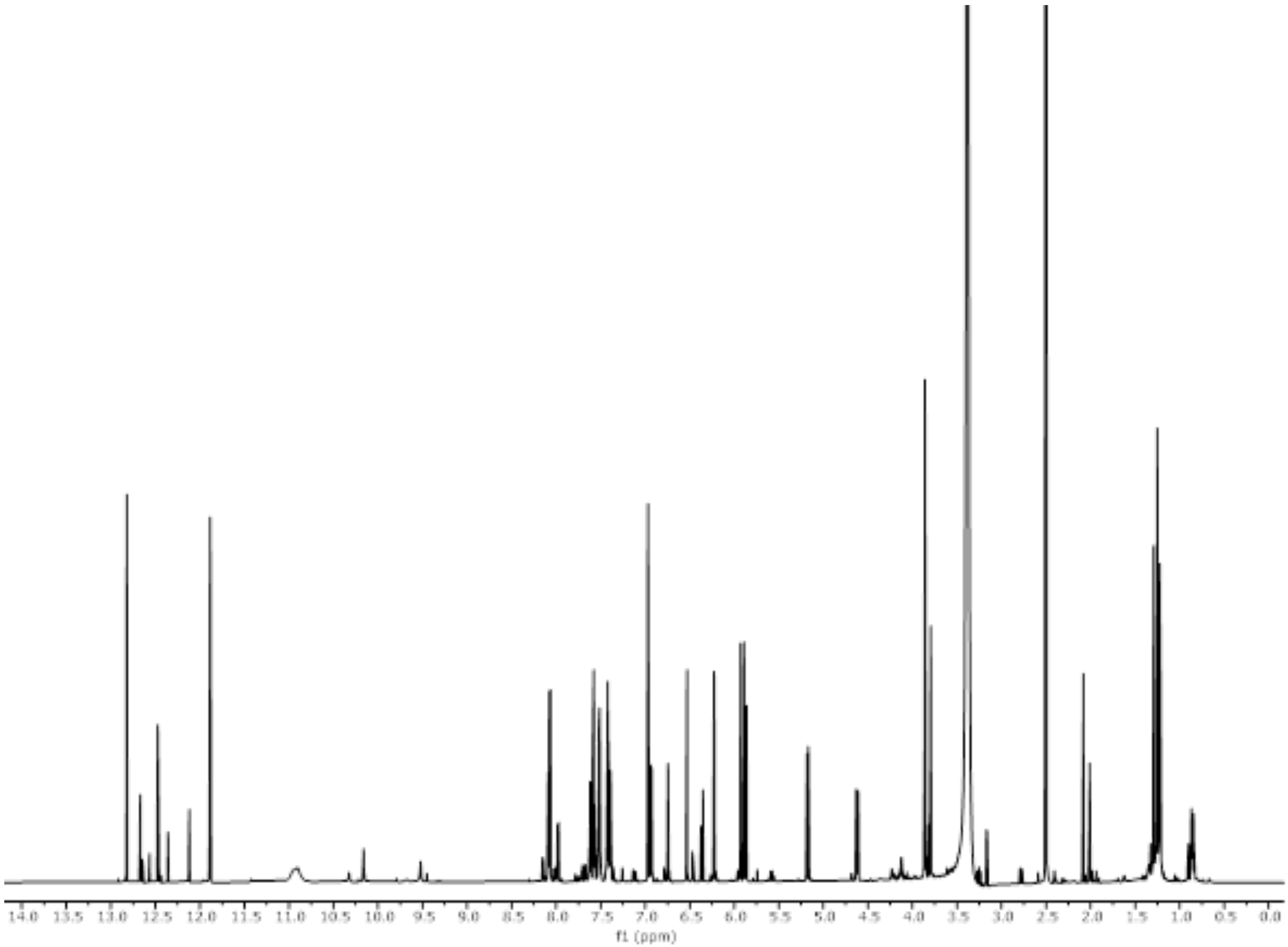

Figure S8. ^{1}H NMR spectrum of F101 obtained from fractionation of the methanol extract of propolis (700 MHz, DMSO-d6).

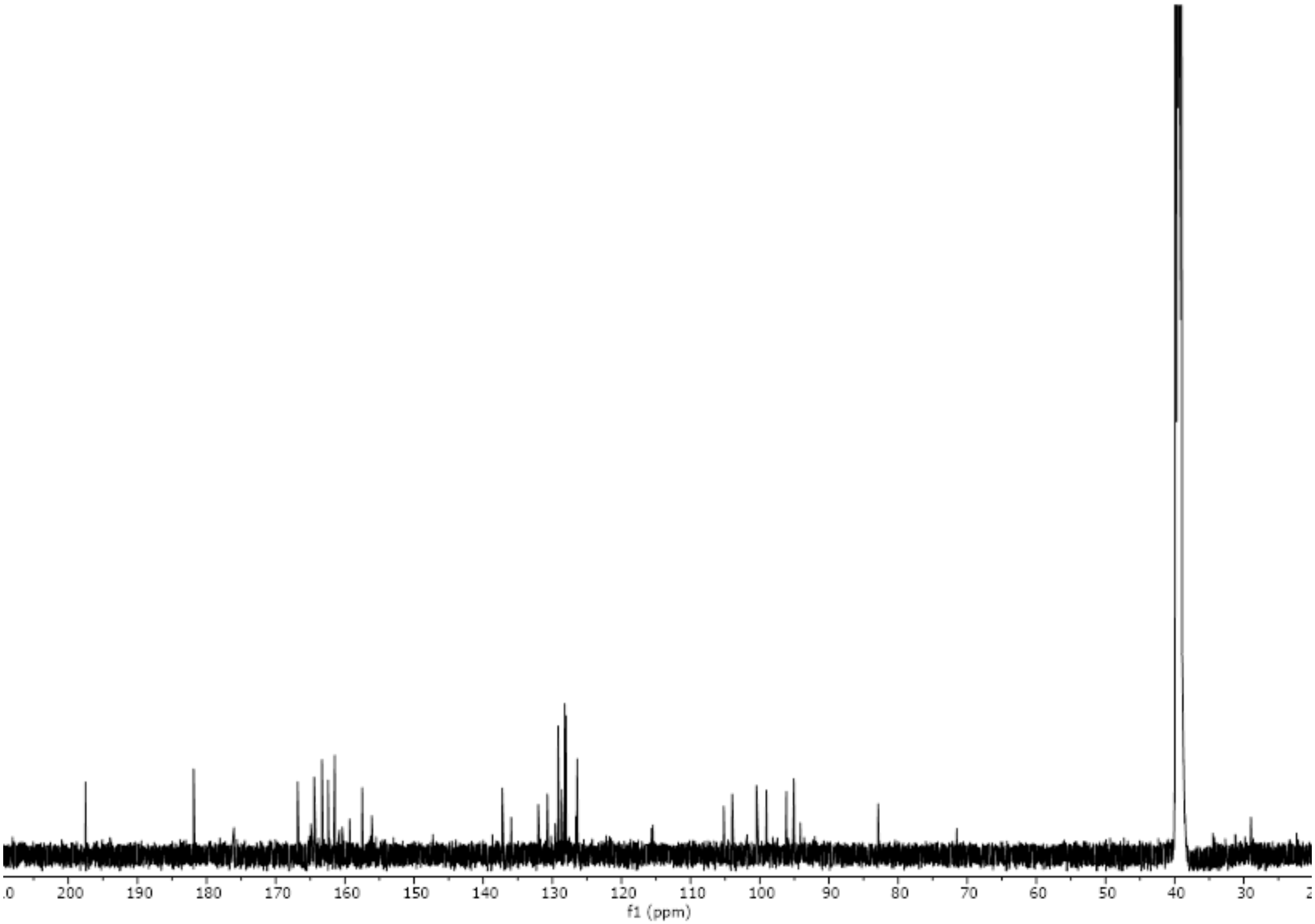

Figure S9. ^{13}C NMR spectrum of F101 obtained from fractionation of the methanol extract of propolis (150 MHz, DMSO-d6).

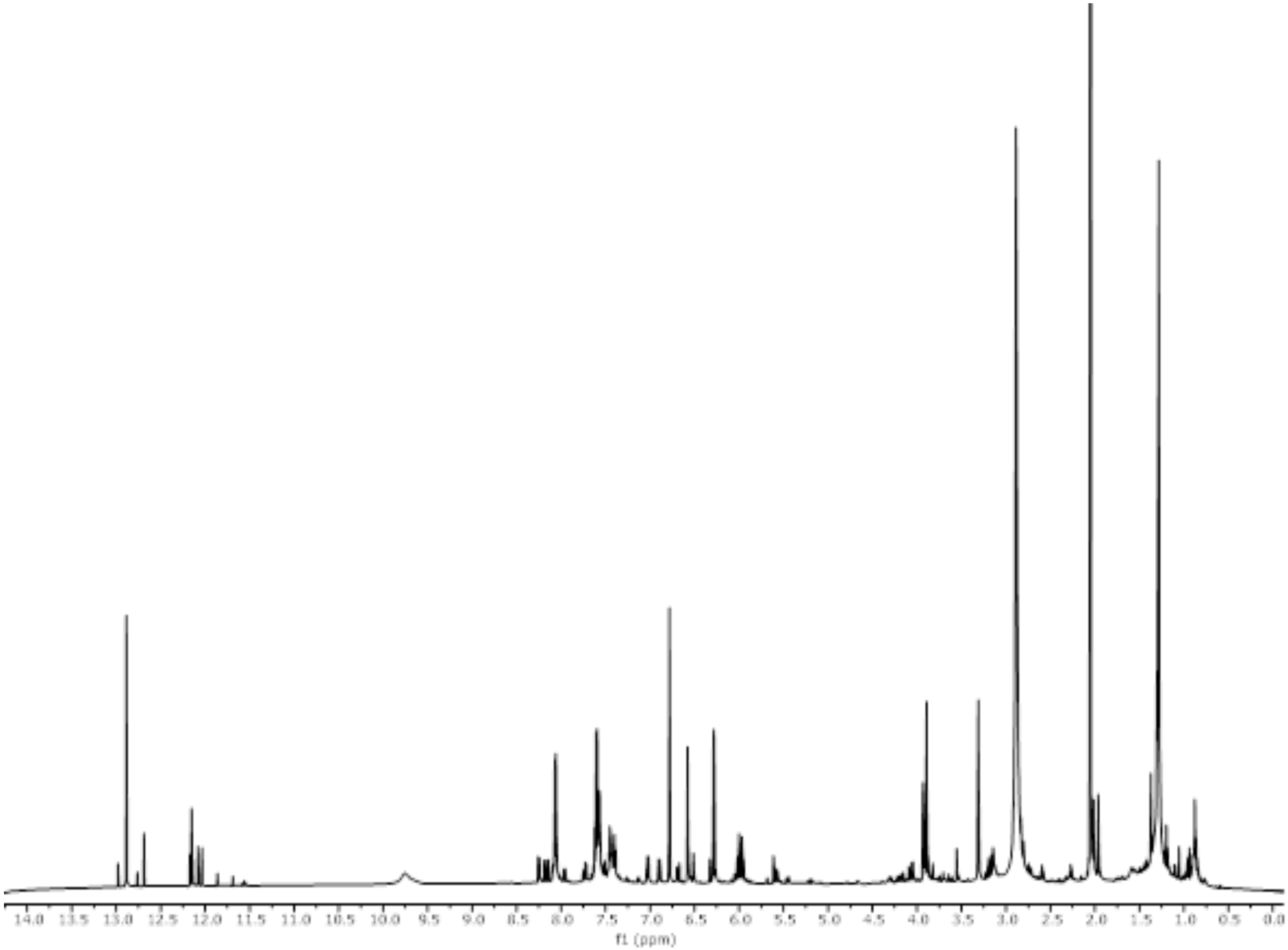

Figure S10. ^{1}H NMR spectrum of F130 obtained from fractionation of the methanol extract of propolis (600 MHz, Acetone-d6).

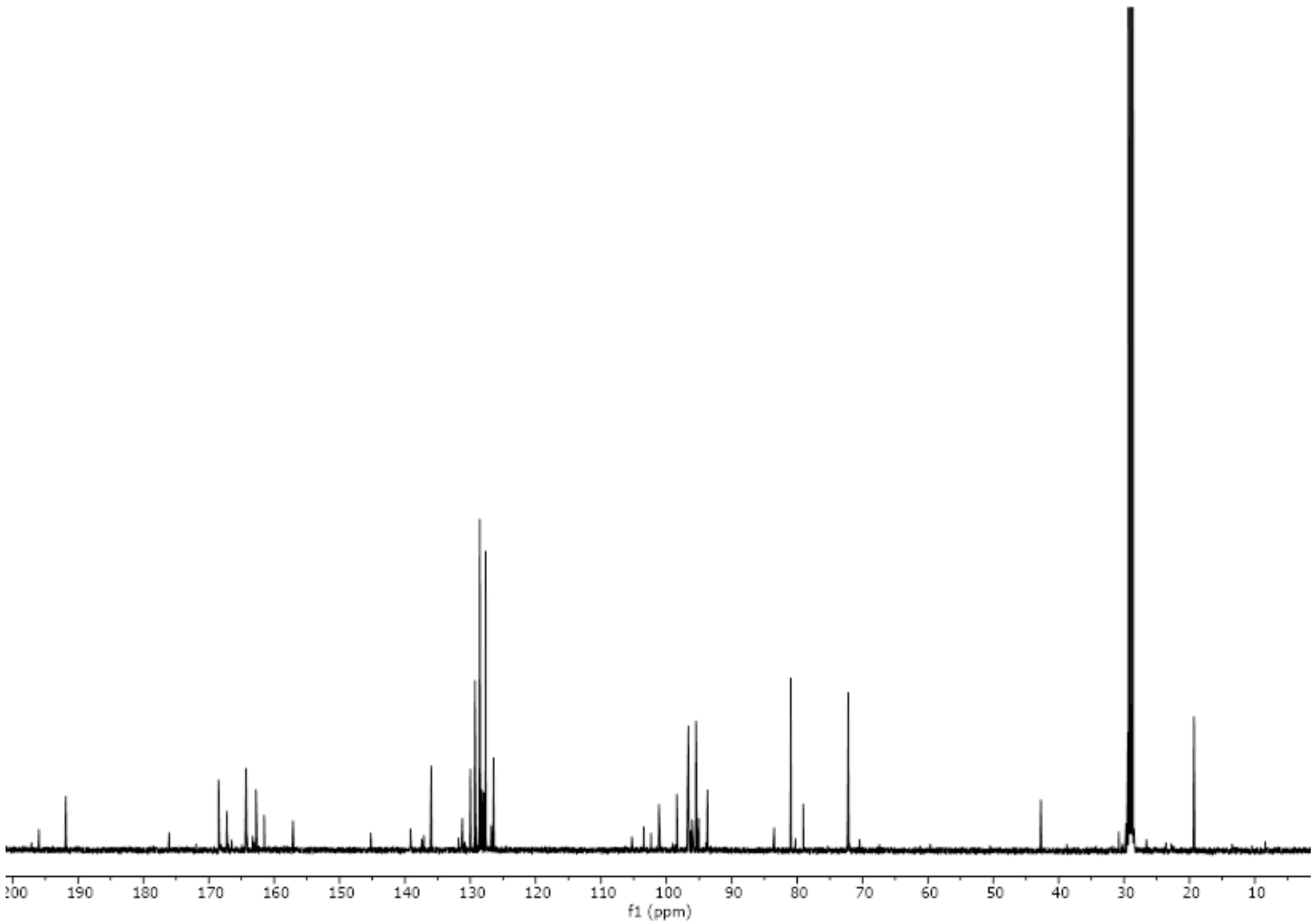

Figure S11. ^{13}C NMR spectrum of F130 obtained from fractionation of the methanol extract of propolis (150 MHz, Acetone-d6).

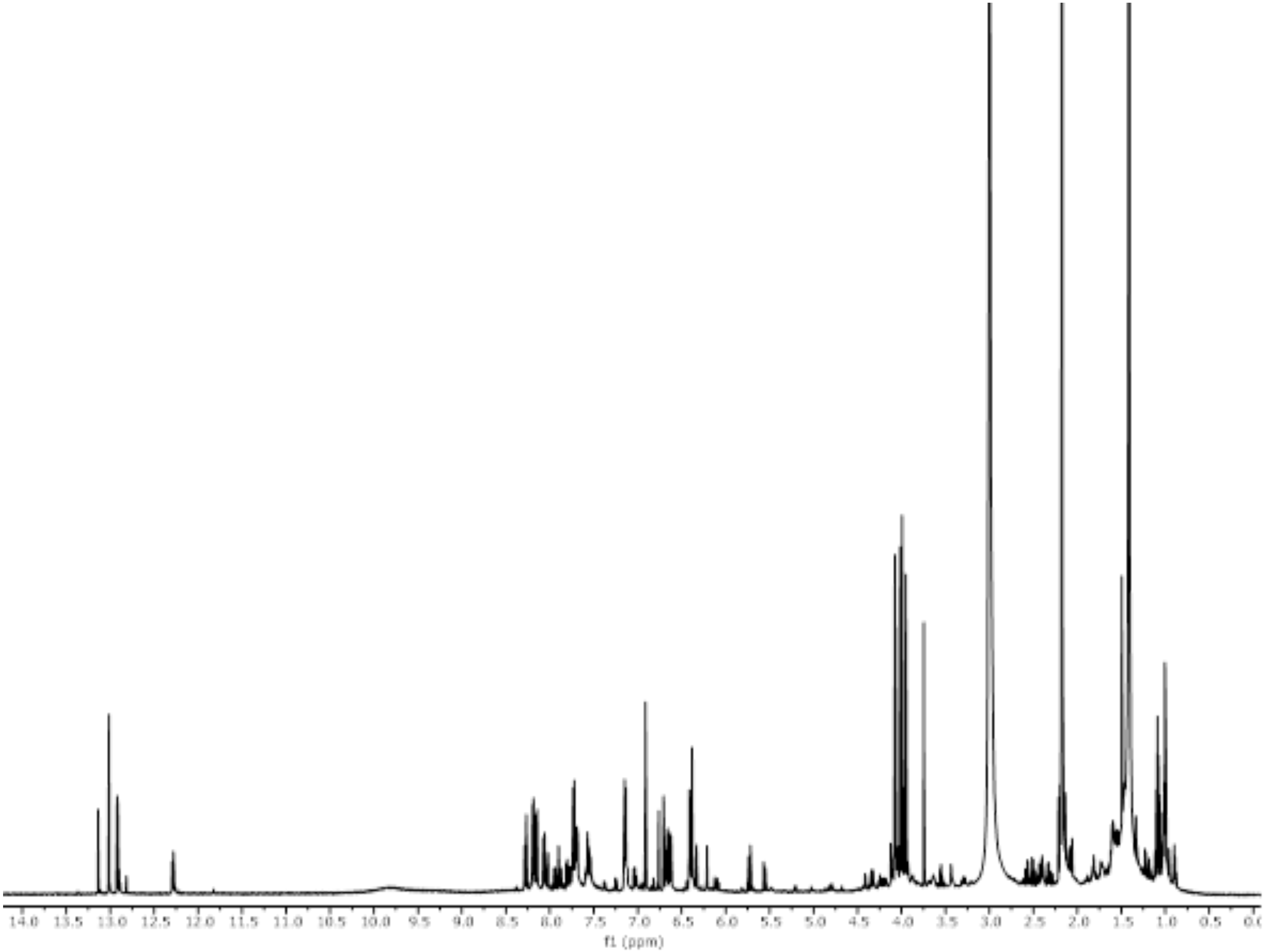

Figure S12. ^{1}H NMR spectrum of F160 obtained from fractionation of the methanol extract of propolis (600 MHz, Acetone-d6).

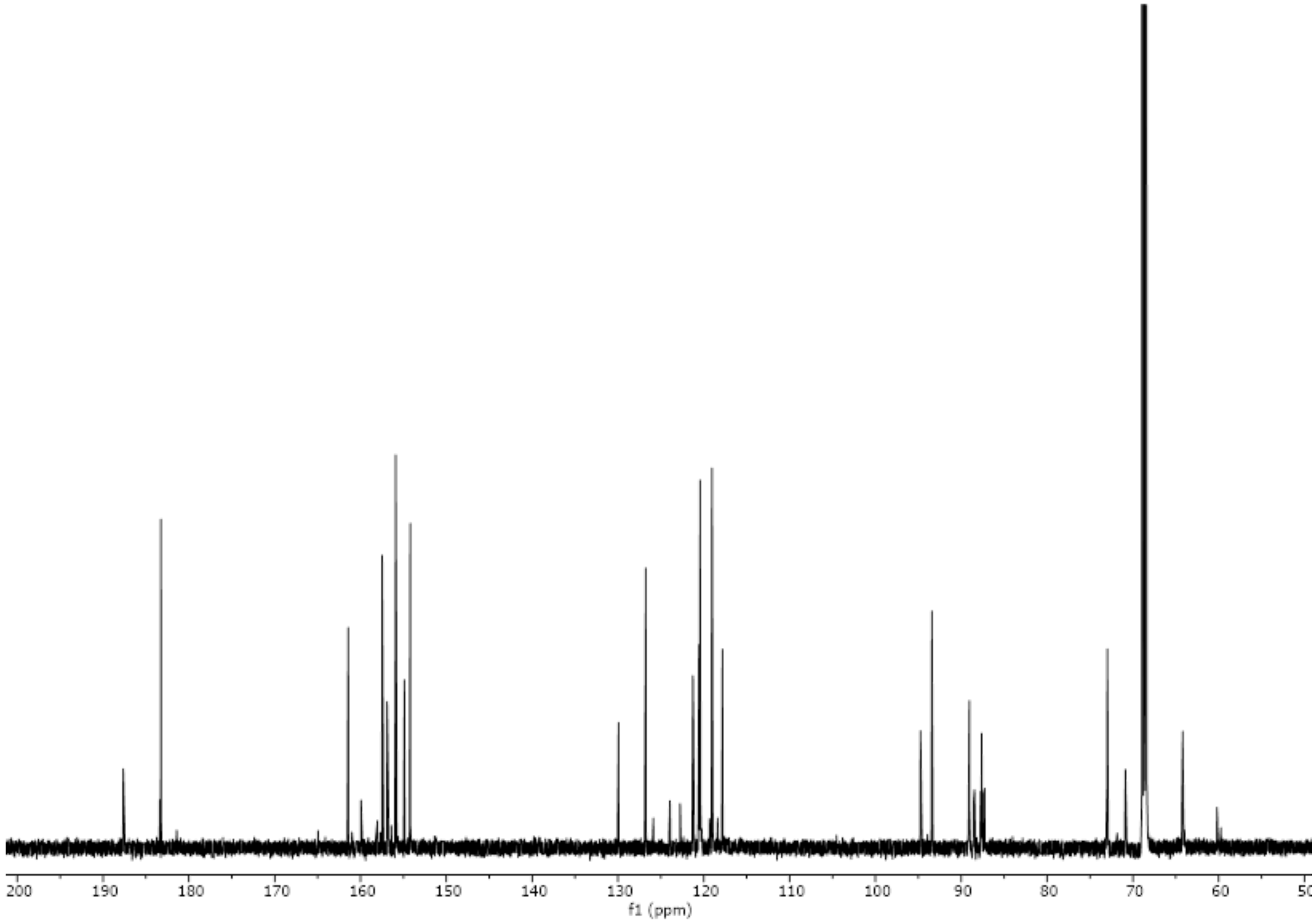

Figure S13. ^{13}C NMR spectrum of F160 obtained from fractionation of the methanol extract of propolis (150 MHz, Acetone-d6).

Display Report

Analysis Info

Analysis Name	D:\Data\Elena Vargas\042822_Propoleo_Neg.d	Acquisition Date	28/04/2022 12:25:45 p.m.
Method	tune_Low negative.m	Operator	Daniel Arrieta
Sample Name	042822_Propoleo_Neg	Instrument	micrOTOF-Q 228888.10392
Comment			

Acquisition Parameter

Source Type	ESI	Ion Polarity	Negative	Set Nebulizer	0.4 Bar
Focus	Active	Set Capillary	3500 V	Set Dry Heater	180 °C
Scan Begin	50 m/z	Set End Plate Offset	-500 V	Set Dry Gas	4.0 l/min
Scan End	3000 m/z	Set Collision Cell RF	200.0 Vpp	Set Divert Valve	Source

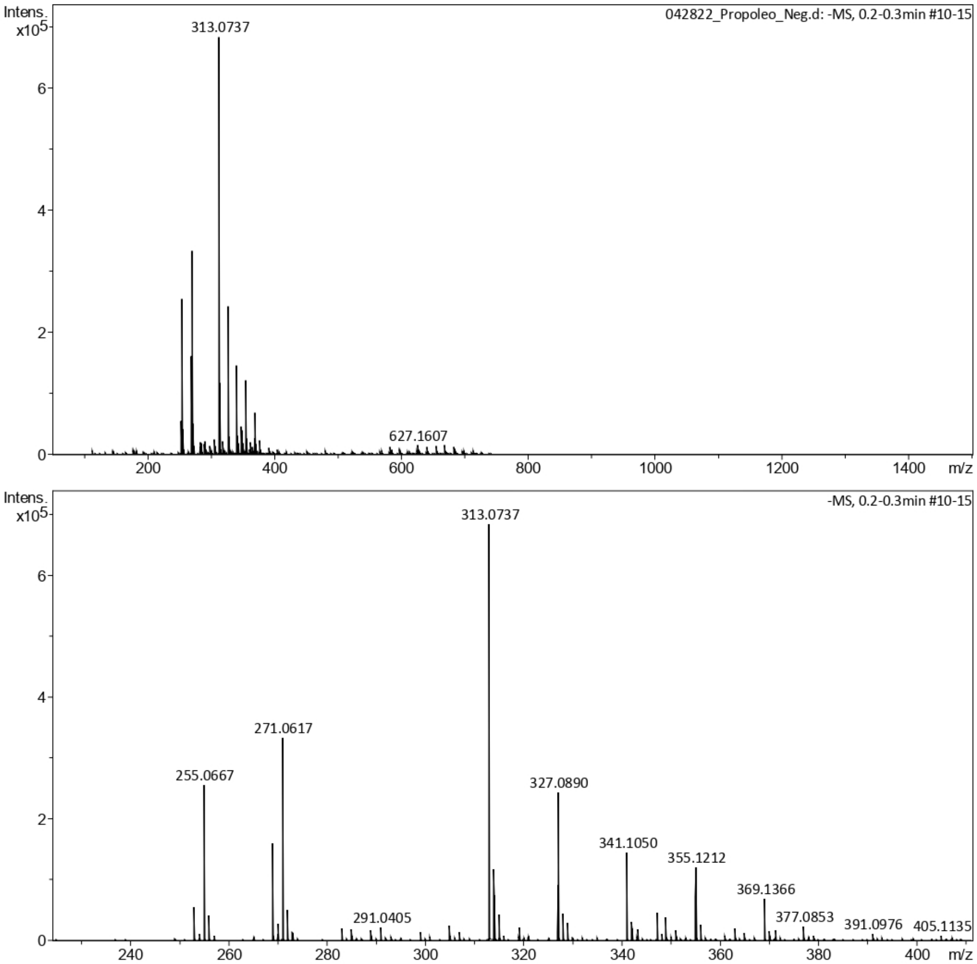

Figure S14. HRMS (ESI-negative mode) spectra of the methanol extract of propolis.

Display Report

Analysis Info

Analysis Name	D:\Data\Elena Vargas\061722_F40_Neg.d	Acquisition Date	17/06/2022 04:20:46 p.m.
Method	tune_Low negative.m	Operator	Daniel Arrieta
Sample Name	061722_F40_Neg	Instrument	micrOTOF-Q 228888.10392
Comment			

Acquisition Parameter

Source Type	ESI	Ion Polarity	Negative	Set Nebulizer	0.4 Bar
Focus	Active	Set Capillary	3500 V	Set Dry Heater	180 °C
Scan Begin	50 m/z	Set End Plate Offset	-500 V	Set Dry Gas	4.0 l/min
Scan End	3000 m/z	Set Collision Cell RF	200.0 Vpp	Set Divert Valve	Source

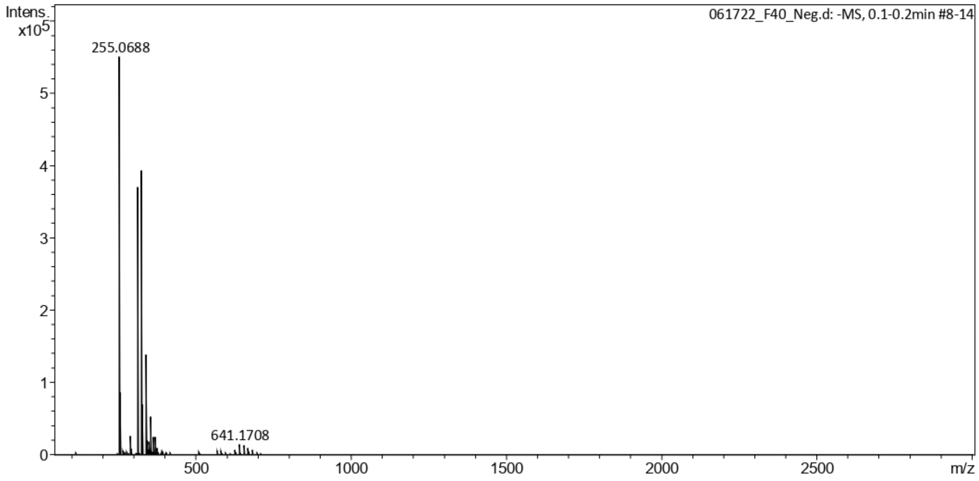

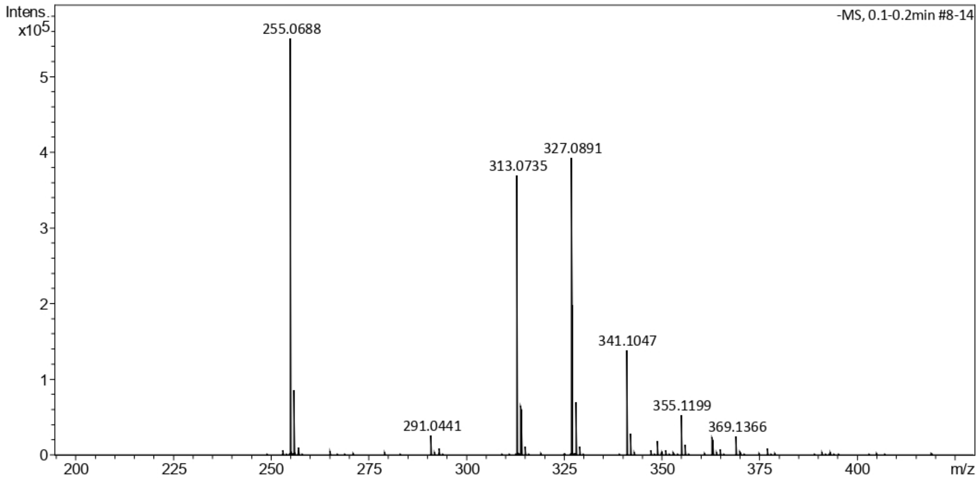

Figure S15. HRMS (ESI-negative mode) spectra of F40.

Display Report

Analysis Info

Analysis Name	D:\Data\Elena Vargas\061722_F80_Neg.d	Acquisition Date	17/06/2022 04:29:21 p.m.
Method	tune_Low negative.m	Operator	Daniel Arrieta
Sample Name	061722_F80_Neg	Instrument	micrOTOF-Q 228888.10392
Comment			

Acquisition Parameter

Source Type	ESI	Ion Polarity	Negative	Set Nebulizer	0.4 Bar
Focus	Active	Set Capillary	3500 V	Set Dry Heater	180 °C
Scan Begin	50 m/z	Set End Plate Offset	-500 V	Set Dry Gas	4.0 l/min
Scan End	3000 m/z	Set Collision Cell RF	200.0 Vpp	Set Divert Valve	Source

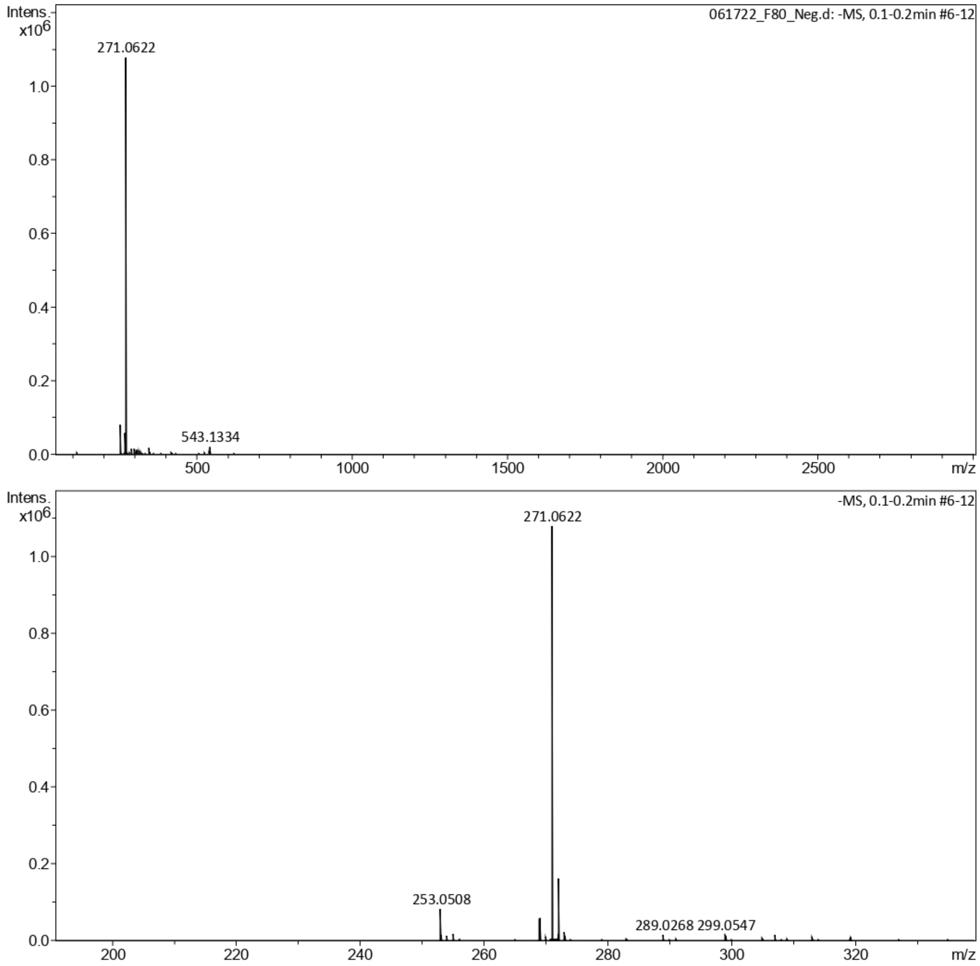

Figure S16. HRMS (ESI-negative mode) spectra of F80.

Display Report

Analysis Info

Analysis Name	D:\Data\Elena Vargas\061722_F101_Neg.d	Acquisition Date	17/06/2022 04:32:53 p.m.
Method	tune_Low negative.m	Operator	Daniel Arrieta
Sample Name	061722_F101_Neg	Instrument	micrOTOF-Q 228888.10392
Comment			

Acquisition Parameter

Source Type	ESI	Ion Polarity	Negative	Set Nebulizer	0.4 Bar
Focus	Active	Set Capillary	3500 V	Set Dry Heater	180 °C
Scan Begin	50 m/z	Set End Plate Offset	-500 V	Set Dry Gas	4.0 l/min
Scan End	3000 m/z	Set Collision Cell RF	200.0 Vpp	Set Divert Valve	Source

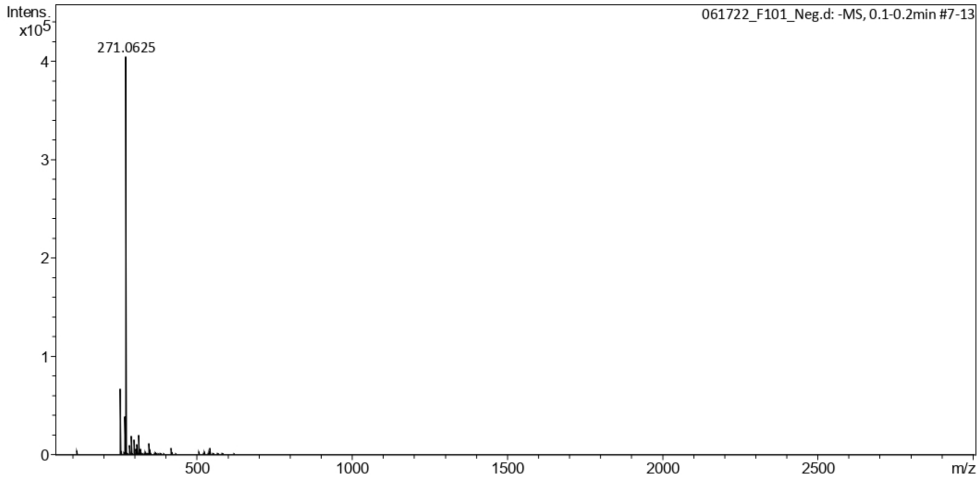

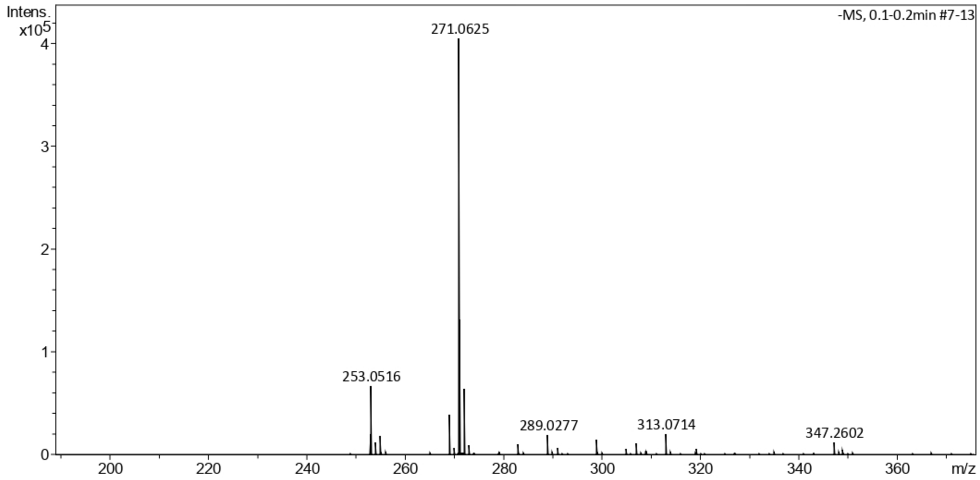

Figure S17. HRMS (ESI-negative mode) spectra of F101.

Display Report

nalysis Info

nalysis Name	D:\Data\Elena Vargas\061722_F130_Neg.d	Acquisition Date	17/06/2022 04:36:55 p.m.
lethod	tune_Low negative.m	Operator	Daniel Arrieta
ample Name	061722_F130_Neg	Instrument	micrOTOF-Q 228888.10392
omment			

cquisition Parameter

ource Type	ESI	Ion Polarity	Negative	Set Nebulizer	0.4 Bar
ocus	Active	Set Capillary	3500 V	Set Dry Heater	180 °C
can Begin	50 m/z	Set End Plate Offset	-500 V	Set Dry Gas	4.0 l/min
can End	3000 m/z	Set Collision Cell RF	200.0 Vpp	Set Divert Valve	Source

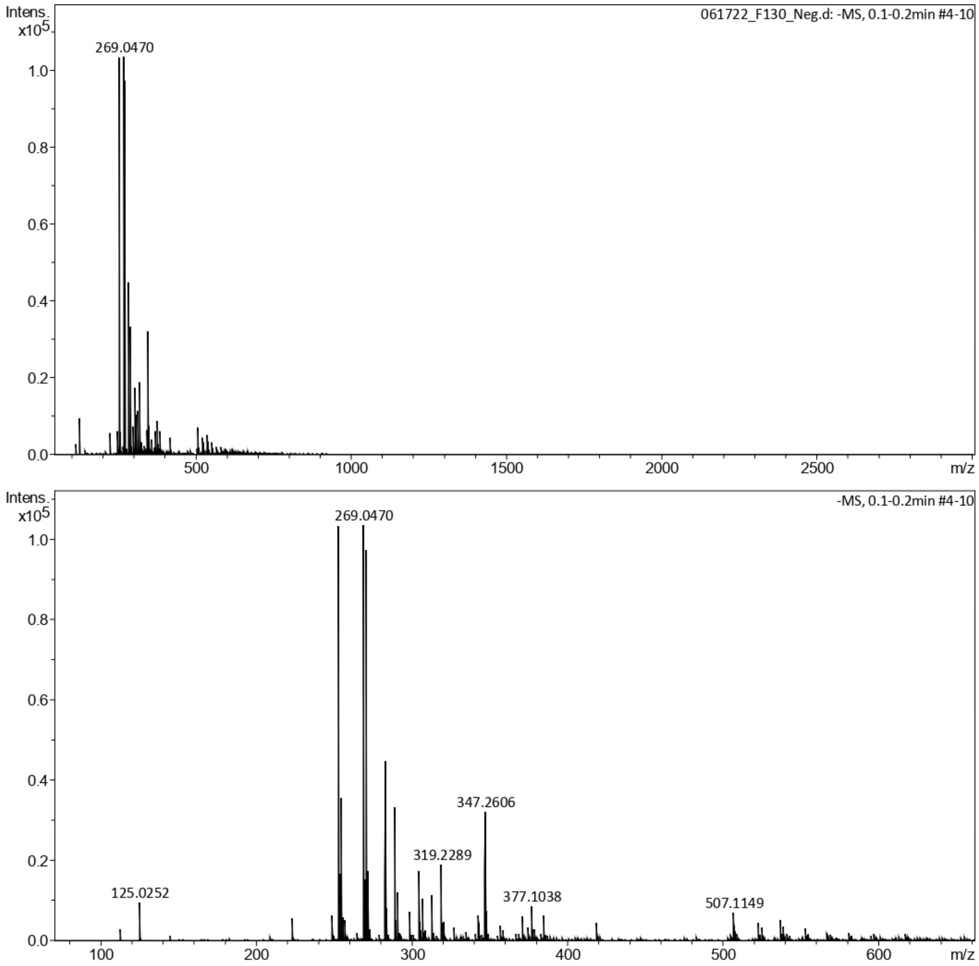

Figure S18. HRMS (ESI-negative mode) spectra of F130.

Display Report

Analysis Info

Analysis Name	D:\Data\Elena Vargas\061722_F160_Neg.d	Acquisition Date	17/06/2022 04:40:28 p.m.
Method	tune_Low negative.m	Operator	Daniel Arrieta
Sample Name	061722_F160_Neg	Instrument	micrOTOF-Q 228888.10392
Comment			

Acquisition Parameter

Source Type	ESI	Ion Polarity	Negative	Set Nebulizer	0.4 Bar		
Focus	Active	Set Capillary	3500 V	Set Dry Heater	180 °C		
Scan Begin	50 m/z	Set End Plate Offset	-500 V	Set Dry Gas	4.0 l/min		
Scan End	3000 m/z	Set Collision Cell RF	200.0 Vpp	Set Divert Valve	Source		

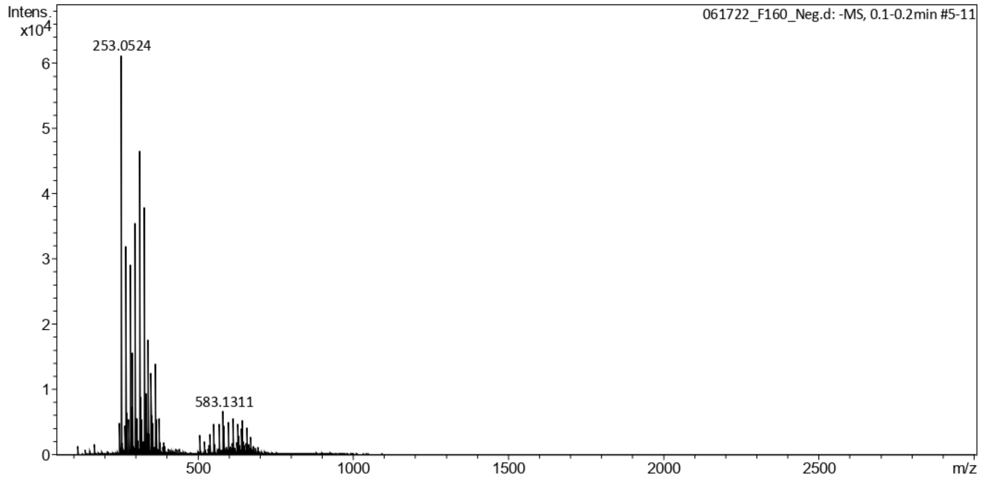

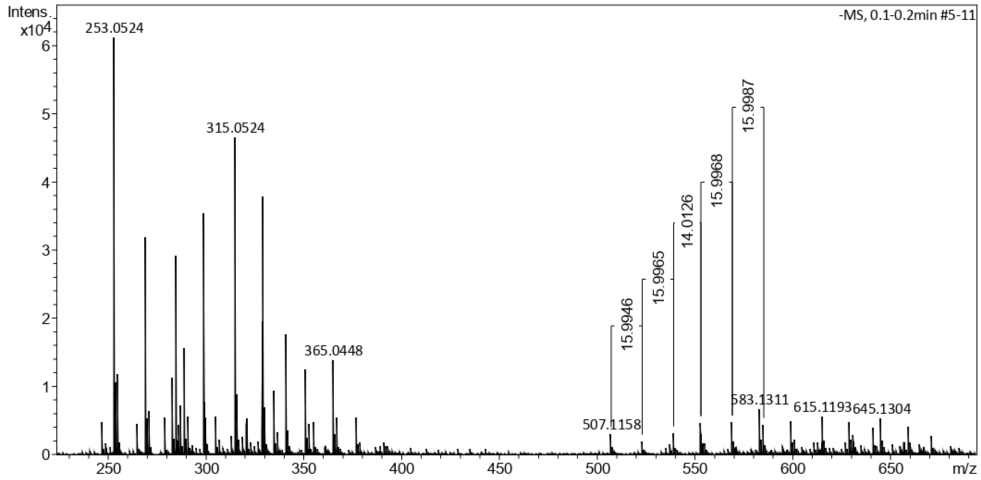

Figure S19. HRMS (ESI-negative mode) spectra of F160.
